# Oncogene-Induced Senescence Transcriptomes Signify Premalignant Colorectal Adenomas

**DOI:** 10.3390/cimb47040221

**Published:** 2025-03-25

**Authors:** Sofian Al Shboul, Heyam Awad, Anas Abu-Humaidan, Nidaa A. Ababneh, Ashraf I. Khasawneh, Tareq Saleh

**Affiliations:** 1 Department of Pharmacology and Public Health, Faculty of Medicine, The Hashemite University, Zarqa 13133, Jordan; 2 Department of Pathology, Microbiology and Forensic Medicine, School of Medicine, The University of Jordan, Amman 11942, Jordan; h_awad@ju.edu.jo (H.A.); a.abuhumaidan@ju.edu.jo (A.A.-H.); 3 Cell Therapy Center, The University of Jordan, Amman 11942, Jordan; n.ababneh@ju.edu.jo; 4 Department of Microbiology, Pathology and Forensic Medicine, Faculty of Medicine, The Hashemite University, Zarqa 13133, Jordan; ashrafkh@hu.edu.jo; 5 Department of Laboratory Medicine, National Institutes of Health Clinical Center, Bethesda, MD 20892, USA; 6 Department of Pharmacology & Therapeutics, College of Medicine & Health Sciences, Arabian Gulf University, Manama P.O. Box 26671, Bahrain

**Keywords:** oncogene-induced senescence (OIS), senescence-associated secretory phenotype (SASP), colorectal cancer, adenoma, single-sample gene set enrichment analysis (ssGSEA)

## Abstract

**Background:** Oncogene-induced senescence (OIS) is a tumor-suppressive mechanism that halts uncontrolled cell proliferation in premalignant lesions. Further investigation into its role in colorectal tumorigenesis is essential. We investigated the expression of OIS transcriptomic landscapes in premalignant colorectal adenomas and whether their resolution is part to adenoma-to-carcinoma progression. **Methods:** Using a publicly available gene expression dataset (GSE117606), we analyzed 66 paired (matched) adenoma–adenocarcinoma samples. Single-sample gene set enrichment analysis (ssGSEA) was performed to assess OIS and senescence-associated secretory phenotype (SASP) signatures, and differential gene expression analysis was conducted to examine key senescence-related genes. **Results:** OIS and SASP signatures were significantly enriched in adenomas compared to adenocarcinomas (*p* < 0.05). Pairwise comparisons confirmed that 65% of patients exhibited higher OIS scores in adenomas, while SASP enrichment declined in 59–61% of cases. Several senescence regulators (*CDKN1A*, *CDKN2B*, and *E2F3*), ECM remodeling genes (*MMP10* and *TIMP2*), and NF-κB-driven SASP factors (*CCL2*, *CXCL2*, *NFKB1*, and *NFKB2*) were significantly downregulated in adenocarcinomas, indicating the resolution of senescence-associated inflammatory signaling during tumor progression. **Conclusions:** These findings support the predominance of OIS phenotypes in colorectal adenomas, suggesting their potential role as a temporary barrier to tumorigenesis in colorectal cancer.

## 1. Introduction

Senescence is a unique cell stress response that commits cells into a terminal growth-arrested state [1]. Senescent cells are also characterized by several biological changes that collectively account for their distinct phenotypic presentation [2], such as enlarged, neuronal-like morphology [3], and exhibiting a reduced nucleocytoplasmic ratio [4]. Despite maintaining a metabolically active state, senescent cells develop dysregulated energetics and mitochondrial dysfunction, and accumulate reactive oxygen species [5]. This is usually accompanied by macromolecular damage to nucleic acids [6,7], proteins [8], and lipids [9]. Furthermore, senescent cells develop enhanced lysosomal activity marked by the upregulation of the senescence-associated β-galactosidase enzyme (SA-β-gal) [10]. Senescent cells undergo broad gene expression alterations [11,12], which account for their ability to secrete a spectrum of soluble and insoluble factors into the microenvironment, collectively termed the senescence-associated secretory phenotype (SASP) [13].

Despite being classically precipitated by replicative exhaustion, senescence can be prematurely induced by stressful stimuli [14], including oncogenic hyperstimulation (oncogene-induced senescence, OIS) [15]. This form of senescence largely accounts for the distinct tumor suppressive nature of senescence which is mediated by its antiproliferative drive, thereby serving as a barrier against malignant transformation [16]. More importantly, the reversal (or evasion) of the senescent growth arrest can accelerate tumorigenesis [17,18,19,20], which partly provides a rationale for the accumulation of OIS cells in premalignant lesions [19,21,22,23,24,25,26,27,28]. However, this has not been demonstrated in colonic tumor models. The tumorigenesis of malignant lesions of the colonic mucosa represents a classical model of the progression from precancerous to cancerous lesions [29]. Colonic adenomas, both villous and serrated, are frequently identified through routine screening colonoscopies where they are typically removed to reduce morbidity, mortality and risk for malignant transformation. Interestingly, mutations leading to the hyperactivation of the proto-oncogenes *K-RAS* and *BRAF* are major drivers of OIS, especially *K-RAS*, which is the classical oncogene utilized experimentally to induce OIS [30]. Accordingly, it is highly likely that OIS is induced as a component of the adenoma–carcinoma transformation process and identified in colonic polyps. Moreover, evidence of OIS is expected to be lacking in fully transformed lesions of adenocarcinoma, as escape from OIS is a step required for precancerous cells to transform into cancer [31].

We investigated whether OIS markers can be identified in colorectal adenomas and compared their expression levels to colorectal adenocarcinoma. We also analyzed senescence-related pathways and gene expression patterns to determine if OIS is actively maintained in adenomas and whether its resolution is observed upon malignant transformation.

## 2. Materials and Methods

### 2.1. Dataset Acquisition and Preprocessing

Gene expression data from GSE117606 was obtained from the Gene Expression Omnibus (GEO) database [32]. The dataset consists of transcriptomic profiles of 66 matched pairs of colon adenocarcinomas and adenomas (each pair was obtained from the same patient), along with an extra 8 adenocarcinomas and 3 adenomas collected as single samples. Raw expression data were processed using the Affymetrix Human Genome U133+ platform (GPL25373), and probe-to-gene mapping was performed using the corresponding annotation file. For genes with multiple probes, the highest expression value was retained.

### 2.2. The Senescence-Associated MSigDB Gene Collections

Three senescence-associated gene sets were obtained from the Molecular Signatures Database (MSigDB), a widely used repository of curated gene sets designed for pathway-based gene expression analysis, as gmt files and were employed for ssGSEA analysis directly [33,34]. To assess senescence-related pathways, we utilized three senescence-specific gene expression signatures from the Molecular Signatures Database (MSigDB), namely, REACTOME_ONCOGENE_INDUCED_SENESCENCE (comprising 35 genes) [35], REACTOME_SENESCENCE_ASSOCIATED_SECRETORY_PHENOTYPE_SASP (comprising 111 genes) [35], and WP_SENESCENCEASSOCIATED_SECRETORY_PHENOTYPE_SASP (comprising 112 genes) [36]. All three signatures were employed to evaluate the presence of OIS and SASP activity in both colorectal adenomas and adenocarcinomas. WP_SENESCENCEASSOCIATED_SECRETORY_PHENOTYPE_SASP has one extra gene (*H1AZ2*) that was not included in REACTOME_SENESCENCE_ASSOCIATED_SECRETORY_PHENOTYPE_SASP. The REACTOME_ONCOGENE_INDUCED_SENESCENCE overlaps by 12 genes with both SASP gene signatures (Supplementary Table S1).

### 2.3. Single-Sample Gene Set Enrichment Analysis (ssGSEA)

To assess the activity of senescence-related pathways, single-sample gene set enrichment analysis (ssGSEA) was performed using the GSEAPY package in Python (version 3.12.7) within the Spyder environment (version 5.5.1). The analysis was conducted on the processed gene expression matrix, where genes were ranked within each sample and compared against predefined gene sets. An enrichment score was assigned per gene set, reflecting its activation level in each sample. ssGSEA calculates enrichment scores by ranking genes within each sample and comparing them against curated gene sets. The scores are then normalized to generate the final normalized enrichment score (NES) for each sample, representing the activity level of a specific MSigDB signature. The resulting NES values were used to compare pathway activity across adjacent mucosa, adenoma, and adenocarcinoma samples.

### 2.4. Statistical Analysis

To determine whether pathway activity differed between adenoma and adenocarcinoma samples, statistical tests were conducted based on sample availability. We used the Shapiro–Wilk test to check for normality. If data followed a normal distribution, a two-sample *t*-test was used for independent samples. If data distribution was non-normal, a Mann–Whitney U test was applied as a non-parametric alternative. When matched adenoma and adenocarcinoma samples from the same patients were available, a paired *t*-test was conducted, and if normality assumptions were violated, a Wilcoxon signed-rank test was used instead. A *p*-value < 0.05 was considered statistically significant, and multiple testing correction was not applied, as the analysis focused on a single senescence-related signature set, eliminating the need for false discovery rate (FDR) correction.

## 3. Results

### 3.1. OIS and SASP Transcriptomic Signatures Predominate in Colorctal Adenomas in Comparison to Their Malignant Counterparts

**Figure 1:**
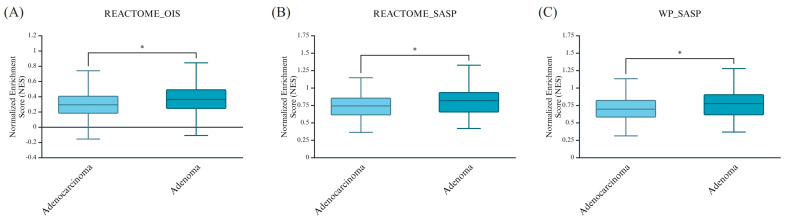
Normalized enrichment score (NES) analysis of OIS and SASP signatures in adenomas and adenocarcinomas. Boxplots represent the NES values for three senescence-related gene sets across adenocarcinoma and adenoma samples. (**A**) The REACTOME_OIS signature shows significantly higher enrichment in adenomas compared to adenocarcinomas (*p* < 0.05). (**B**) REACTOME_SASP and (**C**) WP_SASP signatures follow a similar trend, indicating that senescence and SASP activity were more pronounced in adenomas and declined upon malignant transformation. Statistical significance was determined using *p* < 0.05 (*).

Firstly, we performed single-sample Gene Set Enrichment Analysis (ssGSEA) on all sample types in the dataset. Our ssGSEA analysis revealed higher NES values in adenomas compared to adenocarcinomas for the OIS signature (mean: 0.3844 vs. 0.3066; *p* = 0.022) (Figure 1A). A similar trend was observed when analyzing the two SASP gene collections: REACTOME_SASP (adenoma = 0.805 vs. adenocarcinoma = 0.741, *p* = 0.045) (Figure 1B) and WikiPathways_SASP (adenoma = 0.766 vs. adenocarcinoma = 0.700, *p* = 0.041) (Figure 1C).

**Figure 2:**
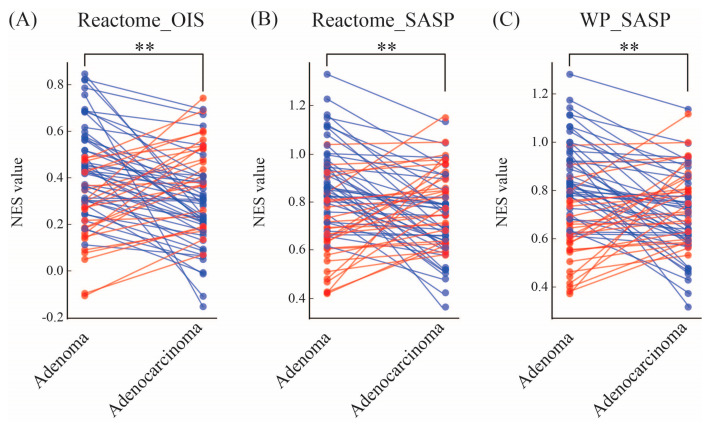
Paired analysis of NES for senescence signatures in colorectal adenomas and adenocarcinomas. Each line represents a paired patient sample (*n* = 66) comparing NES values between adenomas and their matched adenocarcinomas. (**A**) REACTOME_OIS, (**B**) REACTOME_SASP, and (**C**) WP_SASP signatures all exhibit significantly higher enrichment in adenomas compared to adenocarcinomas (** *p* < 0.01). Red lines indicate higher expression (upregulation) in adenocarcinoma relative to adenoma, while blue lines indicate higher expression in adenoma relative to adenocarcinoma.

To further investigate these findings, we examined the NES values in the 66 paired patient samples (adenoma and adenocarcinoma from the same individual). We found that 65% of patients exhibited higher NES values for the OIS signature in adenomas compared to their matched adenocarcinomas (Figure 2A). A similar pattern was observed for SASP signatures, with 59% of patients showing higher NES values for REACTOME_SASP and 61% for WP_SASP in adenomas (Figure 2B,C and Table 1). These findings suggest that senescence transcriptomes are likely to be a component of the premalignant colorectal lesions.

### 3.2. Cell Cycle-Related Genes Are Downregulated Through Malignant Progression of Colorectal Lesions

Next, we performed both bulk (all adenomas vs. all adenocarcinomas) and pairwise (matched for each patient) comparisons at the single-gene level to validate our ssGSEA observations. We selected 19 genes that represent different aspects of cellular senescence with a focus on the senescence-associated growth arrest (SAGA), tissue remodeling, and SASP [37].

**Figure 3:**
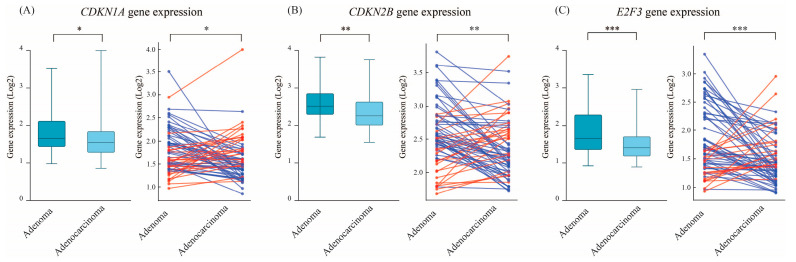
Expression of key senescence-associated cell cycle regulators in adenomas and adenocarcinomas. Boxplots and paired analysis show the expression levels of *CDKN1A* (p21), *CDKN2B* (p15), and *E2F3* in adenomas and adenocarcinomas. (**A**) *CDKN1A*, (**B**) *CDKN2B*, and (**C**) *E2F3* levels were markedly reduced in adenocarcinomas (*p* < 0.001). Paired analysis confirms these trends, with a majority of paired samples exhibiting higher expression in adenomas, reinforcing the role of cell cycle regulators in OIS maintenance. Statistical significance is indicated as *p* < 0.05 (*), *p* < 0.01 (**), and *p* < 0.001 (***). Red lines indicate higher expression (upregulation) in adenocarcinoma relative to adenoma, while blue lines indicate higher expression in adenoma relative to adenocarcinoma.

Among the investigated genes, *CDKN1A*, *CDKN2B*, and *E2F3* are key regulators of the cell cycle and well-established senescence markers [38]. *CDKN1A* expression was significantly higher in adenomas compared to adenocarcinomas (mean: 1.785 vs. 1.637, *p* = 0.041) (Figure 3A, left panel). In the paired analysis, 61% of patients exhibited higher *CDKN1A* expression in adenomas than in their matched adenocarcinomas (*p* = 0.022) (Figure 3A, right panel). Similarly, *CDKN2B* showed a significant decline in expression upon progressing to malignancy, with a mean expression of 2.566 in adenomas vs. 2.333 in adenocarcinomas (*p* = 0.002) (Figure 3B, left panel); these differences were even more pronounced in the paired comparison, where 65% of the adenomas exhibited higher *CDKN2B* expression compared to their matched cancerous tissue (*p* = 0.005) (Figure 3B, right panel). For *E2F3*, mean expression was significantly higher in adenomas (1.81) than in adenocarcinomas (1.49) (*p* = 0.0007) (Figure 3C, left panel). The paired analysis further reinforced this finding with 73% of the 66 paired samples exhibited higher levels of *E2F3* in adenomas (*p* = 0.0001) (Figure 3C, right panel). These results confirm that key SAGA markers are predominant in colorectal adenomas relative to their malignant counterparts.

### 3.3. Senescence-Associated ECM Remodeling Genes Exhibit Lower Expresssion in Colorectal Adenocarcinomas

**Figure 4:**
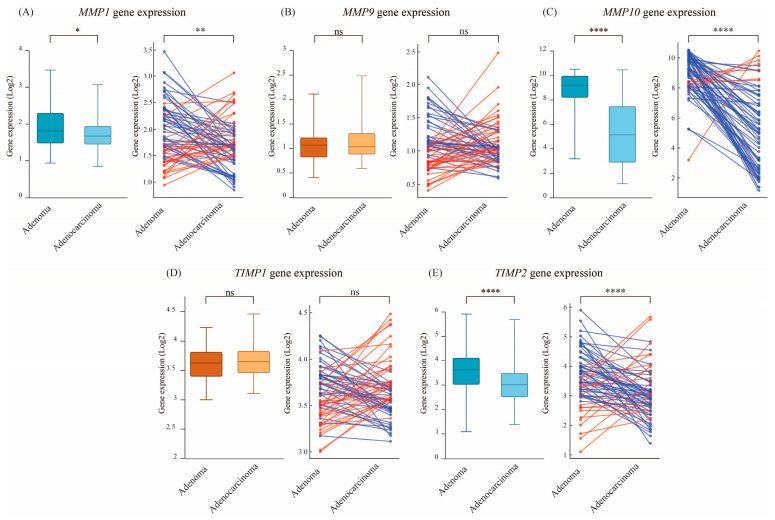
Expression of senescence-associated matrix remodeling genes in adenomas and adenocarcinomas. Boxplots and paired analysis compare the expression of (**A**) *MMP1*, (**B**) *MMP9*, (**C**) *MMP10*, (**D**) *TIMP1*, and (**E**) *TIMP2* in adenomas and adenocarcinomas. These findings suggest that senescence-associated ECM remodeling is more active in adenomas but suppressed upon malignant progression. Red lines indicate higher expression (upregulation) in adenocarcinoma relative to adenoma, while blue lines indicate higher expression in adenoma relative to adenocarcinoma. Statistical significance is indicated as ns: not significant, *p* < 0.05 (*), *p* < 0.01 (**), and *p* < 0.0001 (****).

Next, we examined senescence-associated matrix remodeling and structural reorganization genes, including *MMP1*, *MMP9*, *MMP10*, *TIMP1*, and *TIMP2* (Figure 4). *MMP1* expression was significantly higher in adenomas (mean 1.93) compared to adenocarcinomas (mean: 1.69, *p* = 0.017) (Figure 4A, left panel). This difference was further supported by paired analysis, where 64% of patients exhibited higher *MMP1* expression in adenomas relative to their matched malignant samples (Figure 4A, right panel). In contrast, *MMP9* expression did not show significant differences between adenomas (mean: 1.09) and adenocarcinomas (mean: 1.11, *p* = 0.747) (Figure 4B, left panel), or among the paired comparison (52% of the samples had higher *MMP9* in adenomas, *p* = 0.806) (Figure 4B, right panel). The expression of *MMP10*, however, showed a highly significant decrease in adenocarcinomas (mean: 5.29) compared to adenomas (mean: 8.96, *p* = 0.0001) (Figure 4C, left panel). Pairwise analysis further emphasized this trend, with 89% of paired samples exhibiting higher *MMP10* levels in adenomas (*p* = 0.00001) (Figure 4C, right panel). *TIMP1* expression did not significantly differ between adenomas and adenocarcinomas at the bulk level (Figure 4D, left panel), or in the pairwise comparison (Figure 4D, right panel). Conversely, *TIMP2* showed a significant increase in adenomas (mean: 3.63) compared to adenocarcinomas (mean 3.07, *p* = 0.0001) (Figure 4E, left panel). Paired analysis confirmed this trend, with 73% of paired samples showing a decrease in *TIMP2* expression in adenocarcinomas relative to their matched adenomas (Figure 4E, right panel). These findings indicate that *MMP10* and *TIMP2* are strongly downregulated in adenocarcinomas.

### 3.4. NF-κB and SASP-Related Cytokines Decline upon Progression from Adenoma to Adenocarcinoma Status

**Figure 5:**
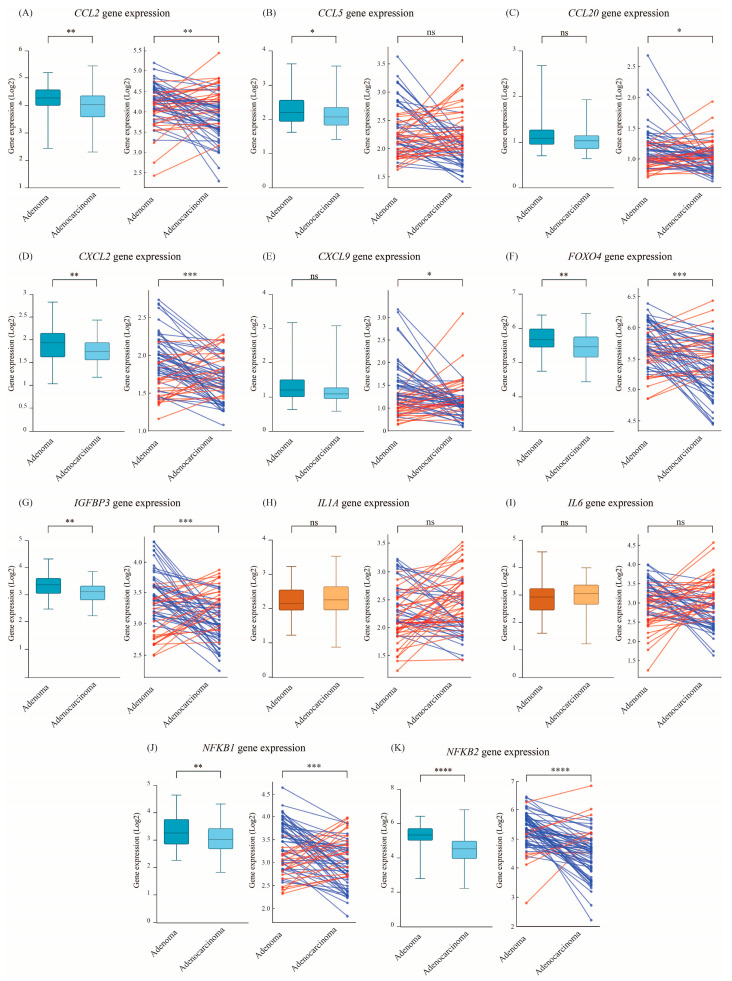
Expression of senescence-associated secretory phenotype (SASP) genes in adenomas and adenocarcinomas. Boxplots and paired analysis illustrate the expression of key SASP-related genes involved in inflammation, immune signaling, and transcriptional regulation in adenomas and adenocarcinomas. (**A**–**C**) Chemokines involved in immune cell recruitment (*CCL2*, *CCL5*, and *CCL20*). (**D**,**E**) Pro-inflammatory cytokines *CXCL2* and *CXCL9*. (**F**,**G**) FOXO4 and IGFBP3. (**H**,**I**) IL6 and IL1A. (**J**,**K**) NF-κB transcriptional regulators (*NFKB1* and *NFKB2*). Red lines indicate higher expression (upregulation) in adenocarcinoma relative to adenoma, while blue lines indicate higher expression in adenoma relative to adenocarcinoma. Statistical significance is indicated as ns: not significant, *p* < 0.05 (*), *p* < 0.01 (**), *p* < 0.001 (***), and *p* < 0.0001 (****).

Finally, we assessed the expression of key SASP factors in adenomas and adenocarcinomas to determine whether SASP-associated inflammation declines during malignant transformation. Several chemokines, cytokines, and transcriptional regulators exhibited differential expression patterns, highlighting potential mechanisms underlying OIS resolution in colorectal tumorigenesis. Chemokines associated with immune cell recruitment showed a marked decline in expression in adenocarcinomas compared to adenomas. *CCL2* expression was significantly higher in adenomas (mean: 4.23) than in adenocarcinomas (mean: 3.97, *p* = 0.002) (Figure 5A, left panel). Pairwise analysis confirmed this trend with 64% of paired samples exhibiting a higher *CCL2* expression in adenomas (*p* = 0.009) (Figure 5A, right panel). Similarly, *CCL5* was significantly downregulated in adenocarcinomas (*p* = 0.035) (Figure 5B, left panel), although this difference was not statistically significant in the paired analysis (53% of adenomas had higher *CCL5* expression, *p* = 0.082) (Figure 5B, right panel). *CCL20* followed a similar pattern, but statistical significance was only observed in the paired analysis (59% of adenomas had higher expression, *p* = 0.031) and not in the bulk comparison (Figure 5C). Among pro-inflammatory cytokines, *CXCL2* expression was significantly lower in adenocarcinomas (*p* = 0.007) (Figure 5D, left panel), with paired analysis confirming this trend (67% of samples exhibited higher expression in adenomas, *p* = 0.0002) (Figure 5D, right panel). *CXCL9* followed a similar trend but was only significantly lower in adenocarcinomas in the paired analysis, where 59% of adenomas had higher *CXCL9* expression (*p* = 0.027) (Figure 5E). Additionally, *FOXO4* exhibited a significant decline in all adenocarcinomas (*p* = 0.001) as well as in 68% of paired samples (*p* = 0.00004) (Figure 5F). Similarly, *IGFBP3* was significantly higher in adenomas relative to adenocarcinomas (3.34 vs. 3.09, *p* = 0.002) (Figure 5G, right panel) with 67% of paired samples showing a decline in *IGFBP3* expression upon progression to malignancy (*p* = 0.002) (Figure 5G, left panel). Interestingly, *IL6* and *IL1A* did not show significant expression differences between adenomas and adenocarcinomas, even in the paired analysis (Figure 5H,I). The NF-κB transcriptional regulators, *NF-κB1* and *NF-κB1* were significantly downregulated in adenocarcinomas (*p* = 0.003 and 0.00001, respectively) (Figure 5J,K, right panels). In paired analysis, *NF-κB1* expression was higher in 68% of adenomas, but this percentage dropped to 32% in adenocarcinomas (*p* = 0.001) (Figure 5J, left panel). Similarly, *NF-κB2* was highly expressed in 86% of adenomas, but its expression decreased sharply to 14% in adenocarcinomas (*p* < 0.0001) (Figure 5K, left panel).

Table 2 shows the differential expression of senescence-associated genes in paired adenoma–adenocarcinoma samples of which *MMP10* exhibited the most substantial fold change (13.372), reinforcing its marked downregulation in adenocarcinoma. While other genes show more modest differences (fold changes ranging from ~0.92 to 1.80), these results still support a general trend of reduced expression in adenocarcinoma, consistent with the observed resolution of OIS and extracellular matrix remodeling signatures.

## 4. Discussion

The tumorigenesis of colorectal epithelium represents a classical model of the progression from premalignancy to malignancy [29]. Colorectal adenomas are benign outgrowths of the colonic mucosa that harbor a potential risk for the progression to invasive adenocarcinoma. The carcinogenesis model proposed by Volgestin describes a series of genetic mutations that occur during the transformation of normal colonic mucosa into adenomas and then into cancer. The most frequently reported mutations involve the genes: Adenomatous polyposis coli (*APC*), *K-RAS*, *BRAF*, Mothers against decapentaplegic homolog 4 (*SMAD4*), *TP53*, and the mismatch repair genes, *MLH1* and *MSH2*. Interestingly, mutations leading to the hyperactivation of the proto-oncogenes *K-RAS* and *BRAF* are major drivers of OIS, especially K-RAS, which is a primary trigger of OIS [30]. Accordingly, it is highly likely that OIS is induced as a component of the adenoma–carcinoma transformation process and identified in colonic polyps. Moreover, evidence of OIS is expected to be lacking in fully transformed lesions of adenocarcinoma, as escape from OIS is critical for malignant treansformation [31].

Early evidence suggested that the expression of p16^INK4a^ is upregulated in BRAF-driven precancerous lesions and lost in invasive serrated carcinomas [39]. Notably, increased p16^INK4a^ expression has been solidly established in pre-neoplastic colonic adenomatous cells which is inversely correlated with the expression of Ki67, a known proliferation marker, which is highly suggestive of senescence [40]. Moreover, the SASP, likely to be generated from oncogene-induced senescent cells accumulating in premalignant colonic adenomas, appears to be associated with driving transformation, consistent with the pro-tumorigenic role of the SASP [41]. For example, IL-8, a main cytokine released as part of the SASP, is upregulated in adenomatous lesions showing increased p16^INK4a^ and decreased Ki67 expression [42].

The gene expression profile of senescent cells is heterogenous [43]. Single-cell sequencing of senescent cells has also revealed that this heterogeneity can exist even within a monoclonal population of cells [44,45]. Unfortunately, senescence-associated gene signature expressions have only been established in human cell lines in vitro [12,46,47,48,49]. While transcriptomic signatures for senescence in vivo have been identified in aging mice, this has not yet been established for human tumor samples. Accordingly, establishing similar profiles in human colonic tumor tissue is yet to be unraveled. A previous study utilized whole-exome sequencing of 20 serrated adenoma samples of independent patients and focused on the identification of mutations in genes implicated in OIS [50]. The study identified mutations in senescence-associated genes, namely, *ATM*, *PIF1*, *TELO2*, *XAF1*, and *RBL1*, in five of twenty subjects with multiple adenomas, indicating germline loss-of-function variants in genes that regulate senescence pathways with the development of serrated adenomas [50].

In this work, we analyzed the transcriptomes of 66 matched colorectal adenoma–adenocarcinoma pairs and uncovered a robust enrichment of OIS signatures in adenomas relative to their malignant counterparts. Specifically, approximately 65% of the patients displayed a higher expression of senescence-related markers (e.g., cyclin-dependent kinase inhibitors, CDKIs) in adenomas, supporting the hypothesis that OIS could be actively engaged in restraining early tumor development. Furthermore, senescence-associated ECM remodeling genes (*MMP1*, *9*, *and 10* and *TIMP1 and 2*) exhibited increased levels in adenomas in relation to their matched malignant tissues where *MMP10* was high in most of the samples. Finally, we found that SASP transcripts were elevated in about 59–61% of adenomas, both using two SASP signatures and a single-gene level that included genes such as *CCL2*, *CXCL2*, *FOXO4*, *and NKFB1 and 2*, highlighting the proinflammatory milieu often accompanying OIS. These data collectively suggest that senescent pathways serve as a key barrier against malignant progression, with their apparent decline in adenocarcinomas likely reflecting a necessary step for full transformation.

These findings align with our earlier investigations of OIS in cervical precancerous lesions, wherein we likewise observed that senescence markers were predominantly expressed in premalignant tissue and diminished as malignancies progressed [51,52]. For example, nearly half of precancerous cervical specimens consistently exhibited the downregulation of lamin B1, a hallmark of OIS [53,54], contrasting sharply with the lower frequency seen in invasive cancer [51]. Another analysis similarly highlighted the enrichment of OIS and SASP factors in cervical intraepithelial neoplasia, a pattern that was notably attenuated in advanced disease [52]. By pointing to a shared senescent landscape in both colorectal and cervical premalignant contexts, these data underscore the broader importance of OIS as an early, tissue-spanning tumor-suppressive mechanism. Recent work in pancreatic lesions demonstrated that selectively targeting senescent cells can hinder the progression of precancerous growth, thus reinforcing the functional significance of OIS in early oncogenesis [55]. Moreover, a comprehensive transcriptomic analysis of senescent fibroblasts found strong involvement of *CDKN1A* (p21^Cip1^) in maintaining the senescent state, a pattern that aligns closely with our results that *CDKN1A* exhibited higher expression in adenomas [56]. p21^Cip1^ serves as a critical cell-cycle inhibitor, reinforcing growth arrest and sustaining the senescent phenotype. Additionally, our analysis highlights a significant upregulation of *NFKB1* and *NFKB2* along with downstream targets, particularly SASP-associated cytokines and chemokines. This is consistent with the findings of Scanlan et al. [56]. These findings reinforce that despite differences in tissue type, NF-κB signaling acts as a shared regulatory axis coupling senescence enforcement with an inflammatory transcriptional program. While the resolution of the OIS-related signatures is indicative of a state of escaping the senescent phenotype, one alternative explanation behind it is that the accumulating senescent, premalignant cells might be a target for removal by the immune system [57,58], especially in the context of colorectal tumorigenesis [15,59].

On the other hand, we cannot ignore the fact that OIS is not necessarily a universal mechanism for adenoma progression to malignancy across all tissue and cell types. One issue along this line is that while the three gene signatures analyzed in this study contain a substantial number of genes (ranging from 35 to 112), this broad inclusion may introduce false positives and reduce specificity in detecting OIS. Prior research indicates that refining gene signatures to a minimal set of key OIS-related genes can improve accuracy, as demonstrated in Ras-Raf-MEK tumors, where filtering out genes associated with other phenotypes, for example, quiescence, reduced background noise and identified five core OIS genes [60]. Additionally, an OIS-positive signature does not inherently pinpoint the specific driver gene mutations responsible for uncontrolled cell proliferation. Instead, a more targeted approach—focusing on a minimal yet precise set of genes directly linked to driver mutations (e.g., *KRAS G12C*) or tumor suppressor gene inactivation (e.g., *APC I1307K* and *TP53* hotspot mutations)—may enhance the specificity and clinical relevance of OIS-based analyses.

While senescent cells can be routinely identified in culture (and to a lesser extent in animal models) based on changes in the expression of several senescence-associated biomarkers, their detection in patients’ samples (human tissue) continues to represent a significant challenge [61]. This is due to several reasons: (*i*) the utilization of the canonical senescence biomarker SA-β-gal is limited to frozen tumor samples (rather than the more readily available fixed tumors), and the use of archived frozen samples (rather than flash frozen, fresh samples) is subject to error, since the activity of SA-β-gal might be altered [62]; (*ii*) the reliance on using a single senescence biomarker, an approach that is not recommended even for in vitro studies [39,40,63]; and (*iii*) the use of markers that are not well-established for senescence [64]. Collectively, these issues largely invite the development and validation of specific senescence-associated transcriptomic signatures that are largely reflective of senescence in vivo [65].

Lastly, our work has several limitations. First, the small sample size limits the ability to establish statistical correlation with the contribution of senescence induction to disease outcome [39,40]. However, our aim from the sample selection in this work was to make sure to maintain a pairwise comparison of colonic lesions with the same genetic background (premalignant vs. malignant) obtained from the same patient, which has definitely led to a limited sample number. Second, this work relied on bulk RNA-seq data, which precludes the resolution of cellular heterogeneity and lacks protein-level validation or functional insights into senescence mechanisms that could possibility be provided through single-cell or spatial transcriptomic analyses [66]. Of note, single-cell analyses in cancer have shown that senescence/OIS can present in multiple “sub-states”, each with distinct transcriptional programs and microenvironmental interactions, revealing, for example, a tendency of certain senescent tumor cell subpopulations to metastasize [67,68]. Finally, the lack of clinical follow-up data hinders the correlation of senescence signatures with patient prognosis (e.g., survival rates and recurrence risk) [69,70].

Accordingly, more comprehensive approaches, including larger cohorts, multi-omics validation (protein/functional assays), spatial/single-cell transcriptomics, protein expression validation, and the integration of clinical outcomes—are sought to overcome such investigational barriers. Future validation efforts should focus on protein-level confirmation of both classical key senescence regulators such as p16^INK4A^, p21^Cip1^, p15^INK4b^, and γH2Ax [71,72,73] and some of the targets identified in this work (e.g., SASP factor *MMP10*) or by others (e.g., SASP factor *MMP7*) in matched colorectal adenomas and carcinomas [68]. If the findings in this work have been validated in large-scale, independent sets, then the resolution of senescence-specific signatures could not only serve as a predictor of a full malignant transformation of colorectal lesions, but could also serve as a biomarker for the potential (not yet considered) utilization of senescence-eliminating therapy (i.e., serotherapy) as a pharmacological approach for the mitigation of the progression of premalignant colorectal lesions [55,74].

## Figures and Tables

**Table 1:** NES comparison of OIS and SASP signatures in paired adenoma–adenocarcinoma samples.

	Higher in Adenoma% (*n*)	Higher in Adenocarcinoma% (*n*)
REACTOME_ONCOGENE_INDUCED_SENESCENCE (NES values)	65% (43)	35% (23)
REACTOME_SENESCENCE_ASSOCIATED_SECRETORY_PHENOTYPE_SASP (NES values)	59% (39)	41% (27)
WP_SENESCENCEASSOCIATED_SECRETORY_PHENOTYPE_SASP (NES values)	61% (40)	39% (26)

**Table 2:** Differential expression of senescence-associated genes in paired adenoma–adenocarcinoma samples (*n* = 66).

		Higher in Adenoma% (*n*)	Higher in Adenocarcinoma% (*n*)	Fold Change
Cell cycle-related genes	*CDKN1A*	61% (40)	39% (26)	1.127
	*CDKN2B*	65% (43)	35% (23)	1.143
	*E2F3*	73% (48)	27% (18)	1.254
Senescence-associated ECM remodeling genes	*MMP1*	53% (35)	47% (31)	1.188
	*MMP9*	48% (32)	52% (34)	0.992
	*MMP10*	89% (59)	11% (7)	13.372
	*TIMP1*	53% (35)	47% (31)	0.986
	*TIMP2*	64% (42)	36% (24)	1.436
NF-κB and SASP-related cytokines	*CCL2*	64% (42)	36% (24)	1.181
	*CCL5*	53% (35)	47% (31)	1.105
	*CCL20*	59% (39)	41% (27)	1.078
	*CXCL2*	67% (44)	33% (22)	1.142
	*CXCL9*	59% (39)	41% (27)	1.136
	*FOXO4*	68% (45)	32% (21)	1.242
	*IGFBP3*	67% (44)	33% (22)	1.207
	*IL1A*	42% (28)	58% (38)	0.925
	*IL6*	55% (36)	45% (30)	1.066
	*NFKB1*	68% (45)	32% (21)	1.256
	*NFKB2*	86% (57)	14% (9)	1.803

## Data Availability

The gene expression dataset analyzed in this study is publicly available in the Gene Expression Omnibus (GEO) under the accession number GSE117606 (https://www.ncbi.nlm.nih.gov/geo/query/acc.cgi?acc=GSE117606, accessed on 11 February 2025). The molecular signature gene sets used for pathway analysis were obtained from the Molecular Signatures Database (MSigDB) (https://www.gsea-msigdb.org/gsea/msigdb/index.jsp, accessed on 11 February 2025).

## Supplementary Materials

The following supporting information can be downloaded at: https://www.mdpi.com/article/10.3390/cimb47040221/s1.

## Abbreviations

The following abbreviations are used in this manuscript:

APC	Adenomatous polyposis coli
CRC	Colorectal cancer
CDKI	Cyclin-dependent Kinase Inhibitor
GEO	Gene Expression Omnibus
MSigDB	Molecular Signatures Database
OIS	Oncogene-induced senescence
SA-β-gal	Senescence-associated-β-galactosidase
SAGA	Senescence-associated growth arrest
SASP	Senescence-associated secretory phenotype
ssGSEA	Single-sample gene set enrichment analysis
