# Supplementary material for: Oncogene-Induced Senescence Transcriptomes Signify Premalignant Colorectal Adenomas
Source: Curr Issues Mol Biol. 2025 Mar 25;47(4):221. doi: 10.3390/cimb47040221 (PMC12026309; doi:10.3390/cimb47040221)
Supplement: Supplementary file 1 [file cimb-47-00221-s001.zip › cimb-3536636-supplementary.pdf]

Table S1. Genes that were used for each of the corresponding signatures (collections). Yellow highlighted genes are shared among the three collections. WikiPathways_SASP and Reactome_SASP differ only in one gene (green highlighted).

REACTOME_ONCOGENE_INDUCED_SENESCENCE	REACTOME_SENESCENCE_ASSOCIATED_SECRETORY_PHENOTYPE_SASP	WP_SENESCENCEASSOCIATED_SECRETORY_PHENOTYPE_SASP
AGO1	ANAPC1	ANAPC1
AGO3	ANAPC10	ANAPC10
AGO4	ANAPC11	ANAPC11
CDK4	ANAPC15	ANAPC15
CDK6	ANAPC16	ANAPC16
CDKN2A	ANAPC2	ANAPC2
CDKN2B	ANAPC4	ANAPC4
CDKN2C	ANAPC5	ANAPC5
CDKN2D	ANAPC7	ANAPC7
E2F1	CCNA1	CCNA1
E2F2	CCNA2	CCNA2
E2F2	CDC16	CDC16
E2F3	CDC23	CDC23
ERF	CDC26	CDC26
ETS1	CDC27	CDC27
ETS2	CDK2	CDK2
ID1	CDK4	CDK4
MAPK1	CDK6	CDK6
MAPK3	CDKN1A	CDKN1A
MDM2	CDKN1B	CDKN1B
MDM4	CDKN2A	CDKN2A
MIR24-1	CDKN2B	CDKN2B
MIR24-2	CDKN2C	CDKN2C
MOV10	CDKN2D	CDKN2D
RB1	CEBPB	CEBPB
RPS27A	CXCL8	CXCL8
SP1	EHMT1	EHMT1
TFDP1	EHMT2	EHMT2
TFDP2	FOS	FOS
TNRC6A	FZR1	FZR1
TNRC6B	H2AB1	H2AB1
TNRC6C	H2AC14	H2AC14
TP53	H2AC18	H2AC18
UBA52	H2AC19	H2AC19
UBB	H2AC20	H2AC20
UBC	H2AC4	H2AC4
	H2AC6	H2AC6
	H2AC7	H2AC7
	H2AC8	H2AC8

	H2AJ	H2AJ
	H2AX	H2AX
		H1AZ2
	H2AZ2	H2AZ2
	H2BC1	H2BC1
	H2BC10	H2BC10
	H2BC11	H2BC11
	H2BC12	H2BC12
	H2BC12L	H2BC12L
	H2BC13	H2BC13
	H2BC14	H2BC14
	H2BC15	H2BC15
	H2BC17	H2BC17
	H2BC21	H2BC21
	H2BC26	H2BC26
	H2BC3	H2BC3
	H2BC4	H2BC4
	H2BC5	H2BC5
	H2BC6	H2BC6
	H2BC7	H2BC7
	H2BC8	H2BC8
	H2BC9	H2BC9
	H3-3A	H3-3A
	H3-3B	H3-3B
	H3C1	H3C1
	H3C10	H3C10
	H3C11	H3C11
	H3C12	H3C12
	H3C13	H3C13
	H3C14	H3C14
	H3C15	H3C15
	H3C2	H3C2
	H3C3	H3C3
	H3C4	H3C4
	H3C6	H3C6
	H3C7	H3C7
	H3C8	H3C8
	H4C1	H4C1
	H4C11	H4C11
	H4C12	H4C12
	H4C13	H4C13
	H4C14	H4C14
	H4C15	H4C15
	H4C16	H4C16
	H4C2	H4C2
	H4C3	H4C3

	H4C4	H4C4
	H4C5	H4C5
	H4C6	H4C6
	H4C8	H4C8
	H4C9	H4C9
	IGFBP7	IGFBP7
	IL1A	IL1A
	IL6	IL6
	JUN	JUN
	MAPK1	MAPK1
	MAPK3	MAPK3
	MAPK7	MAPK7
	NFKB1	NFKB1
	RELA	RELA
	RPS27A	RPS27A
	RPS6KA1	RPS6KA1
	RPS6KA2	RPS6KA2
	RPS6KA3	RPS6KA3
	STAT3	STAT3
	UBA52	UBA52
	UBB	UBB
	UBC	UBC
	UBE2C	UBE2C
	UBE2D1	UBE2D1
	UBE2E1	UBE2E1
	UBE2S	UBE2S
	VENTX	VENTX
